# Supplementary material for: Murine modeling of menstruation identifies immune correlates of protection during Chlamydia muridarum challenge
Source: bioRxiv. 2024 May 23:2024.05.21.595090. Preprint. [Version 1] doi: 10.1101/2024.05.21.595090 (PMC11142139; doi:10.1101/2024.05.21.595090)
Supplement: Supplement 1 [file NIHPP2024.05.21.595090v1-supplement-1.pdf]

bioRxiv preprint doi: https://doi.org/10.1101/2024.05.21.595090; this version posted May 23, 2024. The copyright holder for this preprint (which was not certified by peer review) is the author/funder, who has granted bioRxiv a license to display the preprint in perpetuity. It is made available under aCC-BY 4.0 International license.

Supplemental Data:

Supplemental Figure 1: The total leukocyte yield from indicated FRT tissue sites is plotted as bar and whiskers graphs over pseudopregnancy and compared with mice administered MPA or mice administered sesame seed oil in the absence of pseudopregnancy as a control. Models used to compare a difference of means were fit using multiple comparisons: p-values with q-values≤0.05 are shown *p≤0.05, **p<0.01, ***p<0.001, ****p<0.0001.

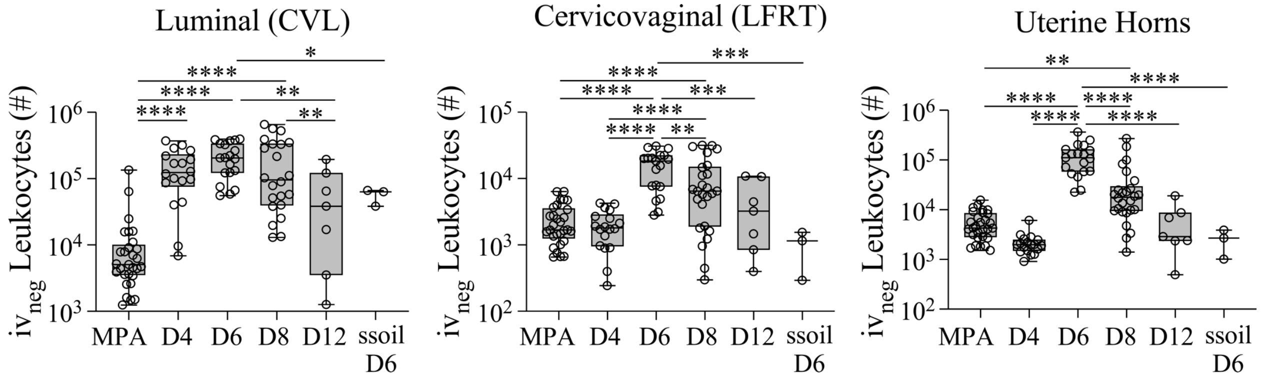

Supplemental Figure 2: An XY graph with prediction bands plotting the ddPCR quantification using dilutions taken from DNA extracted from 1x10^5 IFU of *C. muridarum*. Distributions were tested by Spearman's correlations.

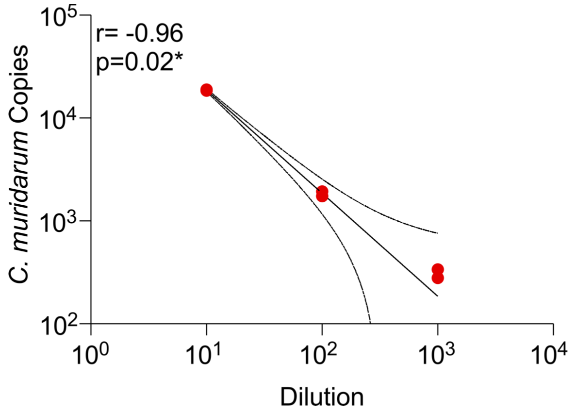

Supplemental Table 1: the mean levels of indicated cytokines and chemokines (pg/mL) with the SEM over pseudopregnancy and in mice administered MPA.

bioRxiv preprint doi: https://doi.org/10.1101/2024.05.21.595090; this version posted May 23, 2024. The copyright holder for this preprint (which was not certified by peer review) is the author/funder, who has granted bioRxiv a license to display the preprint in perpetuity. It is made available under aCC-BY 4.0 International license.

	MPA (pg/mL)	Day 4 (pg/mL)	Day 6 (pg/mL)	Day 8 (pg/mL)	Day 10 (pg/mL)	Day 12 (pg/mL)
IL15	18.825 (3.489)	96.336 (36.655)	218.936 (22.966)	90.755 (24.473)	181.386 (47.04)	32.26 (14.009)
IL-17A	1.242 (0.185)	9.915 (2.939)	80.811 (12.351)	9.481 (2.765)	10.151 (3.025)	2.633 (0.822)
IL-27P28	1.658 (0.421)	4.571 (1.316)	26.045 (4.042)	12.289 (5.05)	16.329 (5.771)	2.016 (0.623)
IL-33	13.544 (4.126)	7.508 (3.059)	5.522 (0.594)	11.091 (3.077)	9.911 (3.03)	1.881 (0.717)
IL-9	0.873 (0.572)	1.856 (.0878)	4.096 (1.016)	1.587 (0.863)	3.234 (2.156)	1 (0.521)
IP-10	8.959 (1.146)	15.375 (4.145)	845.045 (216.52)	103.941 (33.999)	119.646 (49.228)	5.081 (1.767)
MCP-1	2.056 (0.4)	8.631 (4.181)	17.370 (2.771)	19.829 (5.712)	68.625 (42.208)	53.521 (39.849)
MIP-1α	12.803 (1.441)	169.677 (65.429)	329.283 (54.306)	147.607 (67.654)	253.184 (103.611)	24.035 (13.966)
MIP-2	132.679 (21.303)	1188.208 (244.673)	1936 (24.36)	997.099 (250.718)	1461.614 (250.464)	530.557 (202.535)
IFNγ	0.049 (0.012)	0.2 (0.134)	104.177 (20.15)	1.691 (0.599)	16.806 (13.003)	0.201 (0.114)
IL-10	0.733 (0.223)	16.69 (8.08)	64.891 (14.663)	22.646 (9.159)	30.386 (14.889)	0.882 (0.258)
IL12p70	1.339 (0.494)	3.596 (1.177)	30.338 (4.65)	7.966 (3.07)	13.117 (4.122)	1.753 (0.788)
IL-1β	393.715 (55.892)	285.274 (75.729)	1341.195 (174.339)	1163.974 (695.134)	1665.124 (1178.348)	157.367 (63.492)
IL-2	0.038 (0.012)	0.071 (0.017)	0.766 (0.13)	0.599 (0.219)	0.57 (0.253)	0.063 (0.03)
IL-4	0.169 (0.033)	0.089 (0.025)	0.495 (0.092)	0.39 (0.131)	0.249 (0.048)	0.216 (0.076)
IL-5	0.389 (0.056)	0.615 (0.219)	1.867 (0.492)	1.356 (0.527)	1.071 (0.373)	0.14 (0.035)
IL-6	2.16 (0.388)	42.375 (14.848)	7741.421 (1358.467)	714.485 (250.752)	2950.566 (1589.784)	9.655 (5.112)
CXCL1	62.722 (14.326)	137.711 (86.099)	846.746 (138.84)	519.134 (188.805)	882.652 (267.625)	37.582 (12.072)
TNFα	8.714 (0.987)	295.473 (82.585)	2882.011 (357.084)	367.174 (148.467)	371.82 (127.41)	49.419 (20.185)
